# 563 The Why Behind the Release- A 2 Year Review of Pediatric Burn Scar Contracture Releases

**DOI:** 10.1093/jbcr/irad045.159

**Published:** 2023-05-15

**Authors:** Jenna Trost, Creola Woolery, Sarah Bomberger, Karen Robertson, Brett Hartman

**Affiliations:** IU Health, Riley Children's Hospital, Indianapolis, Indiana; Indiana university health Riley children’s hospital, Indianapolis, Indiana; Riley Hospital for Children, Indianapolis, Indiana; IU Health-Riley Hospital for Children, Avon, Indiana; Indiana university/Eskenazi Health, Indianapolis, Indiana; IU Health, Riley Children's Hospital, Indianapolis, Indiana; Indiana university health Riley children’s hospital, Indianapolis, Indiana; Riley Hospital for Children, Indianapolis, Indiana; IU Health-Riley Hospital for Children, Avon, Indiana; Indiana university/Eskenazi Health, Indianapolis, Indiana; IU Health, Riley Children's Hospital, Indianapolis, Indiana; Indiana university health Riley children’s hospital, Indianapolis, Indiana; Riley Hospital for Children, Indianapolis, Indiana; IU Health-Riley Hospital for Children, Avon, Indiana; Indiana university/Eskenazi Health, Indianapolis, Indiana; IU Health, Riley Children's Hospital, Indianapolis, Indiana; Indiana university health Riley children’s hospital, Indianapolis, Indiana; Riley Hospital for Children, Indianapolis, Indiana; IU Health-Riley Hospital for Children, Avon, Indiana; Indiana university/Eskenazi Health, Indianapolis, Indiana; IU Health, Riley Children's Hospital, Indianapolis, Indiana; Indiana university health Riley children’s hospital, Indianapolis, Indiana; Riley Hospital for Children, Indianapolis, Indiana; IU Health-Riley Hospital for Children, Avon, Indiana; Indiana university/Eskenazi Health, Indianapolis, Indiana

## Abstract

**Introduction:**

A burn therapy quality improvement project was identified to determine what factors led to burn scar contracture releases for improved range of motion from June 2020 to June 2022. The goals of the project were to determine: 1) What factors led to the burn scar contracture formation? 2) What factors led to the need for surgery to correct the contractures after the initial injury/surgery? and 3) What therapy interventions could be implemented to identify the patients' contracture risk, improve their outcomes, and prevent additional surgical interventions?

**Methods:**

A review of the burn OR schedule in the EMR was conducted to identify reconstructive surgical cases from June 2020 to June 2022. The following information was obtained on the patients: type of surgery, the location of the release(s), percent TBSA burn, therapy interventions prior to the release, therapy location, and time from initial injury until surgical release.

**Results:**

Out of 52 patients who had surgical interventions for scar contracture: 27 were in the category of patient growth (25 had no limitations in functional AROM at discharge from therapy), 19 in the category of compliance/complex social situations, and 6 in the category of aggressive scarring /difficult scar management. One outlying case was uncategorized.

In the patient growth category, the average time from the initial injury to the release was 8.42 years. In the category of aggressive scarring/difficult scar management, all the patients were under 4 years old and had difficulty with scar management/positioning from the start. The one factor that was noted in both categories was a mVSS score of >/=4 with pliability and vascularity >/=1 and height score of < /=1 at d/c from therapy.

In the compliance/complex social situations category, the factors affecting scar outcome were poor appointment attendance, shared custody, foster system placement, and limited follow-through with home program.4

**Conclusions:**

As a result of this analysis, strategies were implemented to lower the mVSS upon discharge: 1) therapists will set goals for the mVSS score to be < 2 upon discharge to assure that the score is at or below a 1 for pliability and vascularity, and 0 for height; 2) if the mVSS is not < 2 when patient is being considered for d/c from clinic, therapists will discuss this with medical team as patient may be at higher risk for contracture with continued growth; 3) a “maintenance education program” will be provided to all patients at d/c; 4) Social work/psychology will be consulted earlier to work with families on compliance with attendance/interventions; 5) UE casting with new materials utilized by therapists to improve positioning of burned hands/wrists in children < 4 years old.

**Applicability of Research to Practice:**

Therapy practice changes to prevent contractures

